# Community Pharmacist Prescribing: Roles and Competencies—A Systematic Review and Implications

**DOI:** 10.3390/pharmacy13060157

**Published:** 2025-11-01

**Authors:** Stephanie Clemens, Lea Eisl-Raudaschl, Johanna Pachmayr, Olaf Rose

**Affiliations:** 1 Institute of Pharmacy, Pharmaceutical Biology and Clinical Pharmacy, Paracelsus Medical University, 5020 Salzburg, Austria; stephanie.clemens@pmu.ac.at (S.C.); lea.eisl-raudaschl@stud.pmu.ac.at (L.E.-R.); johanna.pachmayr@pmu.ac.at (J.P.); 2 Center of Public Health and Health Services Research, Paracelsus Medical University, 5020 Salzburg, Austria

**Keywords:** pharmacist prescribing, interprofessional care, community pharmacy, international comparison, systematic review, patient safety, implementation barriers

## Abstract

Increasing healthcare demands and physician shortages have prompted many countries to expand clinical responsibilities of pharmacists. Although Canada, the UK, and the US have implemented pharmacist prescribing, other nations lag behind. This review compares international roles, identifies inferred competencies, and explores implications for role expansion. A systematic search of MEDLINE, CINAHL, and the Cochrane Library was conducted using the PICO framework; studies were appraised with Critical Appraisal Skills Programme (CASP) checklists, and interrater reliability assessed via Cohen’s Kappa. Data from 23 studies were thematically synthesized following Preferred Reporting Items for Systematic Reviews and Meta-Analyses (PRISMA) 2020 guidelines. Four themes emerged: (1) expanding clinical and public health roles and pharmacists’ self-perceived readiness; (2) regulatory frameworks defining legal authority, qualifications, and temporary pandemic exemptions; (3) inferred competencies, including micro-skills (patient assessment, guideline application) and macro-capabilities (clinical judgment, accountability, reflective practice); and (4) contextual barriers such as training gaps, limited funding, unclear legal provisions, and workflow challenges. Implementation implications were synthesized and included training, funding, acceptance, and integration. Evidence indicates pharmacist prescribing is safe and patient-centered when supported by regulation, structured training, and systemic integration. Insights from established models can guide incremental implementation, optimizing medication management, enhancing healthcare access, and promoting equitable care.

## 1. Introduction

Global healthcare systems are progressively confronted with challenges arising from demographic changes, an escalating incidence of chronic illnesses, and an anticipated shortage of healthcare personnel, which is expected to exacerbate by the year 2030 [1]. These pressures have driven innovation in workforce roles and service delivery models to improve accessibility and sustainability. One notable development has been the expansion of prescribing rights to community pharmacists in several countries, including the UK, the US, Canada, Australia, Poland, Switzerland, and Denmark [2]. This transition seeks to improve access to primary care, decrease the inappropriate use of emergency department services, and mitigate the workload burden on General Practitioners (GPs), who are experiencing escalating patient demands [1,3].

Pharmacist prescribing constitutes a multifaceted clinical practice that encompasses patient assessment, diagnostic evaluation, formulation of treatment plans, and ongoing monitoring. This role necessitates advanced clinical expertise and proficient decision-making capabilities [4]. The scope of pharmacist prescribing varies internationally, reflecting differences in legal frameworks, professional training, and healthcare system organization. Models range from independent prescribing—where pharmacists autonomously assess and prescribe medications—to supplementary and collaborative prescribing, where prescribing authority is shared or delegated in partnership with physicians [5,6]. Canada has significantly expanded independent prescribing in community settings, while other countries maintain more restrictive or regionally variable prescribing frameworks [6,7]. These differing approaches give rise to continuous discourse concerning the essential competencies, safety considerations, and the most effective incorporation of pharmacist prescribing within healthcare teams.

In addition, practices such as prescribing renewals, therapeutic substitutions, emergency supply, and deprescribing represent important, yet inconsistently regulated, components of pharmacist-led care [2,8]. Despite international progress, no consensus definition or universally accepted regulatory model exists, hindering the transferability of best practices across different contexts [2].

In contrast, pharmacists in many other countries currently provide a broad range of clinical services, including pharmaceutical counselling, medication reviews, point-of-care testing, and compounding. In some countries, like Austria or Poland, pharmacists are legally authorized to dispense emergency prescriptions, provided that specific regulatory conditions are adhered to [9,10,11,12]. Growing shortages of physicians can drive the uptake of prescribing pharmacists, particularly in rural regions [13].

Given this context, pharmacist prescribing could enhance access to care, alleviate the burden on GPs, improve patient outcomes, and make better use of pharmacists’ and physicians’ skills and expertise [6]. However, despite growing international discourse, no comprehensive systematic review has yet compared the prescribing roles and developed inferred competencies, nor explored the implications for healthcare systems.

This systematic review aims to fill this gap by synthesizing international evidence on pharmacist prescribing roles and identifying barriers and facilitators to implementation. The review provides policy-relevant insights to inform future reforms that could leverage pharmacist expertise to improve patient care, ensure safe prescribing practices, and optimize healthcare resource utilization.

## 2. Materials and Methods

This systematic review was conducted following the PRISMA 2020 guidelines to ensure transparency and reproducibility of the research process [14]. The checklist is available in the Supplementary Materials. The review protocol was prospectively registered with the International Prospective Register of Systematic Reviews (PROSPERO) (CRD42025626045) to enhance transparency, prevent duplication, and reduce reporting bias. The studies retrieved from the electronic databases were imported into the reference management software EndNote, which was also used to remove duplicate records.

To enhance the comprehensiveness of evidence retrieval from the databases, the literature search strategy was structured using the PICO framework, which involves formulating the research question by outlining the following components [15]:

- Population (P): Community pharmacists
- Intervention (I): Prescribing roles
- Comparison (C): Traditional roles of pharmacists
- Outcome (O): Barriers and facilitators influencing practice and patient care outcomes, inferred competencies for safe and effective prescribing

Based on the application of the PICO model, the following research questions (RQ) were developed:

- RQ1: What tasks do community pharmacists perform, and in which settings or models is prescribing carried out?
- RQ2: What legal and regulatory frameworks define pharmacists’ prescribing authority, eligibility, and permitted medications?
- RQ3: What barriers and facilitators influence community pharmacists’ prescribing practices and patient care outcomes?
- RQ4: What skills, qualifications, and competencies can be inferred for safe and effective prescribing, and how can these be assessed?

### 2.1. Eligibility Criteria

Inclusion criteria:

- Articles addressing the roles or prescribing practices of community pharmacists;
- Articles published between 2015 and 2025;
- Articles published in English;
- Articles published in peer-reviewed scientific journals;
- Original or primary source studies, including descriptive, experimental, quasi-experimental, cross-sectional, and longitudinal designs.

Exclusion criteria:

- Articles not addressing community pharmacist prescribing or community pharmacist roles;
- Articles published before 2015;
- Articles published in languages other than English;
- Articles published in non-scientific journals, incomplete, or non-peer-reviewed publications;
- Secondary source studies such as reviews, editorials, and commentaries.

### 2.2. Information Sources and Search Strategy

A systematic literature search was conducted across three major bibliographic databases: MEDLINE (via PubMed), CINAHL (via EBSCOhost), and the Cochrane Library (via Ovid), last consulted on 22 January 2025. The strategy was designed to capture studies relevant to community pharmacist prescribing while excluding hospital-based settings. Boolean operators “AND” and “NOT” were employed to combine keywords and refine the search results. The detailed search string for each database retrieved is summarized in Table 1.

### 2.3. Selection Process

To ensure objectivity and minimize potential bias in study selection, a multi-stage screening process was conducted independently by two reviewers (LE, SC) according to the PRISMA statement [14]. Following the removal of duplicate entries, studies were screened in three sequential steps: title screening, abstract evaluation, and full-text review. At each stage, both reviewers independently assessed the studies against the predefined inclusion and exclusion criteria. In instances where discrepancies occurred or agreement could not be reached, a third reviewer (OR) was consulted to resolve the disagreement and provide a final decision. This approach was implemented to enhance the methodological rigor and consistency of the selection process.

### 2.4. Data Collection and Risk of Bias Assessment

Data collection was performed using a structured extraction table to ensure consistency and transparency. For each included study, key characteristics were extracted, including first author and year of publication, country, study design, focus and key area, population and medication(s), and barriers and facilitators.

No effect measures or statistical pooling were applied, owing to the qualitative and descriptive nature of the included studies. Consequently, no sensitivity analyses or meta-analytic techniques were conducted; instead, data were synthesized narratively within a thematically organized framework, ensuring analytical consistency through independent quality appraisal.

The methodological rigor of the final set of included studies was assessed using the Clinical Appraisals Skills Programme (CASP) checklists, selected according to each study design [i.e., cross-sectional, qualitative, or Randomized Controlled Trial (RCT)] [16]. The number of checklist items varied by study type (e.g., 10, 11, 13, or 14 items), reflecting differences across CASP versions tailored to specific study designs. A standardized scoring system was employed, assigning 1 point for “Yes”, 0.5 points for “Can’t tell”, and 0 points for “No” responses of the quality assessments. This facilitated a semi-quantitative evaluation of methodological rigor across the studies. The raw checklist scores were subsequently converted into percentage values to allow for consistent comparison. Based on these percentage scores, studies were classified into quality categories following commonly used thresholds [17]:

- High quality (≥80%)
- Moderate quality (60–79%)
- Low quality (<60%)

To enhance the methodological rigor of the quality appraisal process, a randomly selected subset of 83% of the included studies (19 out of 23) was independently assessed by six independent experts using the same CASP instrument. As a result, each of these studies was reviewed by one of the authors (LE) and one additional person, allowing for direct comparison. Interrater agreement between reviewers was calculated using Cohen’s kappa, as this statistic is appropriate for assessing reliability when two raters evaluate multiple ordinal variables per study [17]. Kappa coefficients were interpreted using the classification by Landis and Koch, 1977, as follows [18]:

- <0.00 = Poor
- 0.00–0.20 = Slight
- 0.21–0.40 = Fair
- 0.41–0.60 = Moderate
- 0.61–0.80 = Substantial
- 0.81–1.00 = Almost perfect

Prior to analysis, an inter-rater reliability range of 0.61 to 0.80 was defined as acceptable, indicating a level of “substantial agreement” [17].

### 2.5. Synthesis Methods

The synthesis of findings was carried out using an inductive coding approach following qualitative synthesis methods [19]. Coding was undertaken independently by two members (SC, LE) of the research team and refined through iterative discussion to ensure consistency and reflexivity. Extracted data were coded line by line and progressively condensed into thematically coherent domains (key themes) aligned with the research questions. Frameworks mentioned in the results section (Cheng et al. and the fishbone diagram) were used to guide the interpretation and organization of themes. Personal and methodological reflexivity were considered throughout the analysis to enhance analytical rigor. A combined narrative and tabular presentation was used to support thematic clarity and facilitate cross-study comparison, which enabled the development of implications for service implementation. If data were unclear or missing, this was noted transparently without assumptions.

### 2.6. Assessment of Reporting Bias and Certainty

Given the small number of studies and high heterogeneity, tests for publication bias were not suitable. Instead, risk of bias was assessed using CASP checklists, and the overall evidence was narratively considered in light of study quality, consistency, and clinical relevance.

## 3. Results

### 3.1. Screening Results

**Figure 1:**
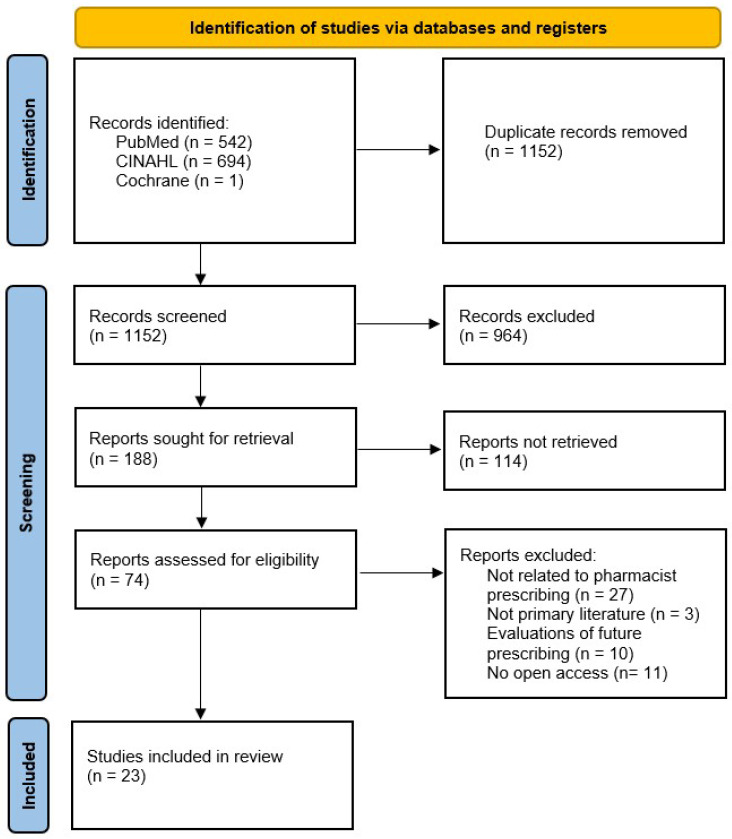
PRISMA 2020 flow diagram of the study selection process.

The search yielded 1152 unique records. Following screening of titles and abstracts, 188 full-text articles were evaluated, resulting in 23 studies being included in the final review. The selection procedure is outlined in the PRISMA flow diagram (Figure 1).

#### Study Characteristics

**Figure 2:**
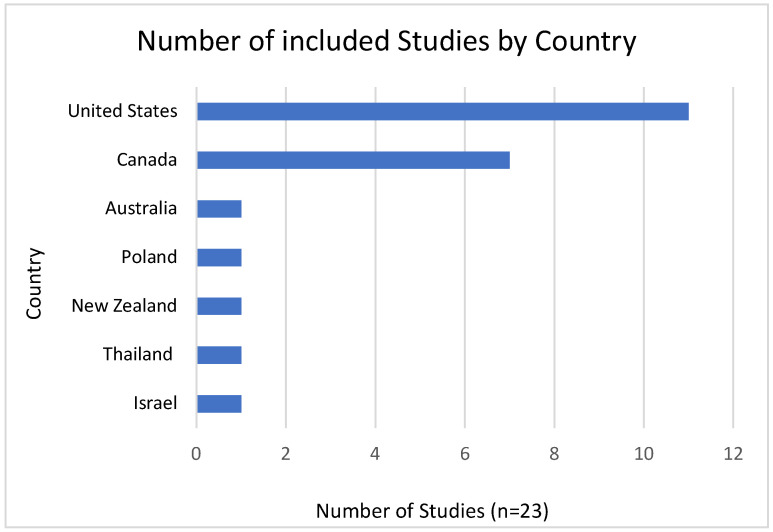
Number of included studies by country (n = 23).

A total of 23 studies, published between 2015 and 2024, met the inclusion criteria and were included in the final review. The key characteristics of these studies are summarized in Table 2. Most studies originated from the United States (n = 11), focusing on pharmacist prescribing for hormonal contraception, statins, naloxone, and Human Papillomavirus (HPV) vaccination [20,21,22,23,24,25,26,27,28,29,30]. Seven studies were conducted in Canada, primarily addressing medications for minor ailments, cardiovascular and metabolic conditions (antihypertensives, lipid-lowering agents, antidiabetics, anticoagulants), opioid agonist therapy, antibiotics, hormonal contraceptives, antifungals, antivirals, antihistamines, smoking cessation aids, GERD treatments, acne therapy, and vaccines [31,32,33,34,35,36,37]. Single studies from Australia, New Zealand, Thailand, Poland, and Israel examined a range of medications including antibiotics, cardiovascular and respiratory agents, dermatological and alimentary tract medications, inhaled corticosteroids and vaccines [38,39,40,41,42]. Figure 2 illustrates the distribution of studies by country (n = 23).

Methodologies included cross-sectional surveys (n = 6), qualitative interviews (n = 6), retrospective/registry/claims analyses (n = 4), RCTs (n = 2), pilot study (n = 1), before-and-after study (n = 1), policy/legal document analyses (n = 2), and mixed-methods study (n = 1). Sample sizes ranged from small qualitative interviews (n = 19–36 pharmacists) to large population-level analyses (n > 370,000 patients), with participants including pharmacists, patients, and regulatory stakeholders.

### 3.2. Risk of Bias Assessment

The methodological quality of the included studies was generally high, with most reporting well-defined objectives, appropriate study designs, and robust participant recruitment strategies. Of the 23 included studies assessed using the Critical Appraisal Skills Programme (CASP) tool, 14 (61%) were rated as high quality, 6 (26%) as moderate, and 3 (13%) as low (Table A1). The number of appraisal items varied (10–14), depending on the study design and corresponding CASP version.

**Figure 3:**
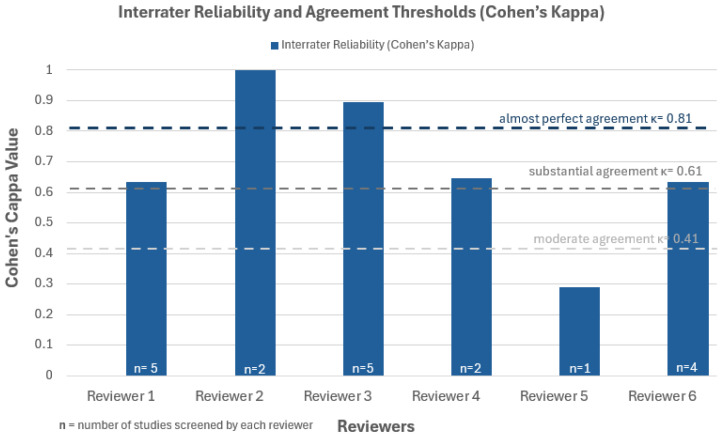
Interrater reliability across 19 included studies, based on Cohen’s Kappa (κ) values and interpreted according to the Landis and Koch (1977) [18] thresholds. Higher κ-values indicate stronger agreement between reviewers.

Interrater agreement was assessed for a subset of 19 studies and is presented in Figure 3. Cohen’s Kappa analysis showed mostly moderate-to-almost-perfect agreement among reviewers, supporting the general consistency of the quality appraisal process, though some variability was observed (Table A2).

### 3.3. Synthesis of Findings (Development of Key Themes)

The inductive coding of the extracted data resulted in four interconnected key themes, which naturally aligned with the focus of the review: the international role of community pharmacists (RQ1), regulatory frameworks (RQ2), contextual barriers and facilitators (RQ3), and inferred competencies (RQ4). Competencies were derived from the roles and frameworks identified across studies, following Cheng et al. (2005), and classified as micro-competencies (job-related) and macro-competencies (person-related), situated within the social and professional context of prescribing pharmacists [43]. Contextual barriers and facilitators were analyzed to capture factors influencing competency enactment across different settings (RQ4). Building on these findings, an Ishikawa (fishbone) diagram was developed to visually synthesize the identified barriers, facilitators, and competencies, thereby illustrating the implications for pharmacist prescribing and serving as a reference point for other countries considering implementation.

**Figure 4:**
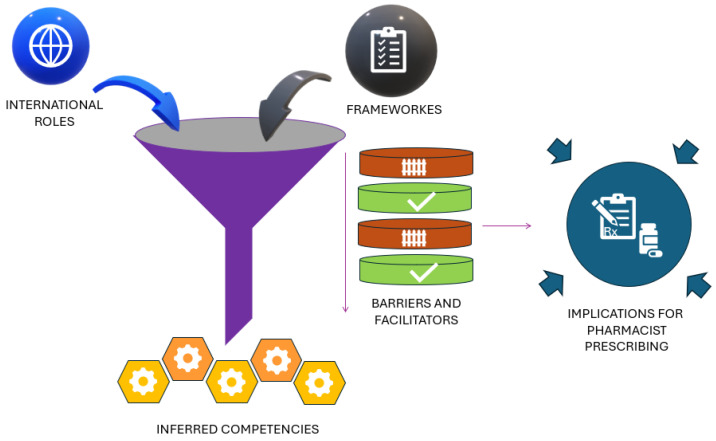
Schematic presentation of key themes that emerged and the development of implications.

Although the themes emerged inductively from the data, their organization reflects the guiding focus of the research questions, providing a coherent structure for integrating evidence and supporting cross-study and cross-country comparison. Findings are presented using a combination of narrative synthesis and tabular summaries to enhance thematic clarity and facilitate systematic comparison. Implications for service implementation were developed based on this synthesis. A schematic overview of the thematic structure is presented in Figure 4.

#### 3.3.1. International Role

Across the included studies, community pharmacists consistently demonstrated an expanded scope of practice that extends well beyond traditional medication dispensing. Three overarching themes emerged to structure these findings: (1) clinical role expansion, (2) public health and accessibility, and (3) readiness and self-perception.

1. Clinical Role Expansion

Several studies highlighted pharmacists’ growing involvement in clinical activities traditionally performed by physicians. These include point-of-care testing, physical examinations, clinical diagnosis, and prescribing based on laboratory results. In Canada, pharmacists using structured test-and-treat models—including risk assessment, education, testing, and prescribing-achieved a 21% greater relative reduction in estimated cardiovascular event risk [36]. Pharmacist-led anticoagulation management and atrial fibrillation monitoring demonstrated high clinical efficacy (treatment and cost efficiency) and safety (stroke risk reduction) [35,37]. In Idaho and New Mexico, pharmacists independently initiated therapies, including statins and contraceptives, with high patient satisfaction and improved access [23,30]. During the COVID-19 pandemic, pharmacists in Canada and Poland played critical roles in medication management and continuity of opioid therapy [33,41]. In Thailand and Australia, pharmacists demonstrated high appropriateness in antibiotic prescribing for common infections, i.e., uncomplicated UTI, cellulitis, adolescent acne [38,40].

2. Public Health and Accessibility

Pharmacists contributed meaningfully to improving access to healthcare, especially in underserved and rural areas: In Canada, pharmacist prescribing reduces the burden on physicians while ensuring high patient satisfaction and effective management of minor ailments such as acid reflux, routine vaccinations, contraceptive care, herpes zoster treatment, and allergic rhinitis [31,34]. In the US, programs in New Mexico and California highlighted pharmacists’ role in addressing access gaps in contraception and opioid overdose prevention [21,25,30]. In Poland and Israel, pharmacists ensured continuity of care during the COVID-19 pandemic by issuing prescriptions and providing vaccinations [41,42].

3. Readiness and Self-Perception

Pharmacists’ perspectives on their preparedness to prescribe varied across contexts. Surveys and interviews revealed generally high confidence in prescribing, particularly where training and regulatory support were in place [22,29]. Pharmacists who had begun prescribing reported enhanced professional identity, satisfaction, and confidence [23,31]. Common difficulties included gaps in formal education, time constraints, and the absence of clear guidelines, especially during early implementation [20,30].

#### 3.3.2. Regulatory Framework

Pharmacist prescribing is fundamentally shaped by national regulatory frameworks, which define legal authority, scope of practice, and qualification requirements. Across the included studies, two main aspects were identified: (1) permanent legal foundations and qualification standards, and (2) temporary exemptions introduced during public health emergencies, such as the COVID-19 pandemic.

1. Legal Authority and Scope

Pharmacists’ prescribing authority and scope of practice differ between countries. In Israel, these rights are linked to professional experience and educational qualifications. Pharmacists with at least two years of practice or a clinical pharmacy degree may dispense previously prescribed medications without a new prescription, using their clinical judgment to ensure uninterrupted essential therapy [42]. These regulations determine not only which medications pharmacists may prescribe but also under which conditions and through which models (e.g., independent versus protocol-based prescribing).

2. Emergency Exemptions and Pandemic-Driven Change

Public health emergencies can prompt temporary modifications of prescribing regulations. During the COVID-19 pandemic, Canada authorized pharmacists to adapt opioid therapy, ensuring continuity of care [33]. Poland expanded emergency prescribing rules to allow longer treatment durations and broader patient eligibility [41]. In some jurisdictions, these temporary changes have become permanent, illustrating regulatory flexibility in response to urgent healthcare needs.

#### 3.3.3. Barriers and Facilitators

Across the included studies, key barriers and facilitators to pharmacist prescribing clustered into four domains: (1) funding, (2) training, (3) integration, and (4) acceptance.

1. Training:

Pharmacists frequently reported gaps in prescribing education, diagnostics, and guideline familiarity, alongside liability concerns [20,22,38]. Prior clinical experience and structured training programs were shown to improve prescribing confidence and readiness [36,40].

2. Funding:

Lack of remuneration and unclear financial incentives consistently limited uptake [20,21,28,30]. Where government funding or cost-effectiveness evidence was available, implementation was more successful [32,37].

3. Acceptance:

Patients reported high trust and satisfaction with pharmacist prescribing across contraception, chronic disease management, and acute conditions [23,30,34]. Remaining barriers included unclear legal protections and stigma [33].

4. Integration:

Workflow disruptions, staffing shortages, and limited access to patient data were common challenges [23,32]. Facilitators included strong digital infrastructure, clear prescribing protocols, and interprofessional collaboration [26,36,41].

#### 3.3.4. Inferred Competencies

Table 3 summarizes community pharmacist prescribing roles, related regulatory frameworks, and the competencies inferred for safe and effective practice. Competencies were classified as macro-competencies (overarching capabilities such as clinical judgment and accountability) and micro-competencies (specific skills such as patient assessment and guideline application). This framework illustrates how pharmacists’ expanded roles are grounded in legal authority and qualification standards, ensuring patient safety and professional accountability.

#### 3.3.5. Implications for Pharmacist Prescribing

**Figure 5:**
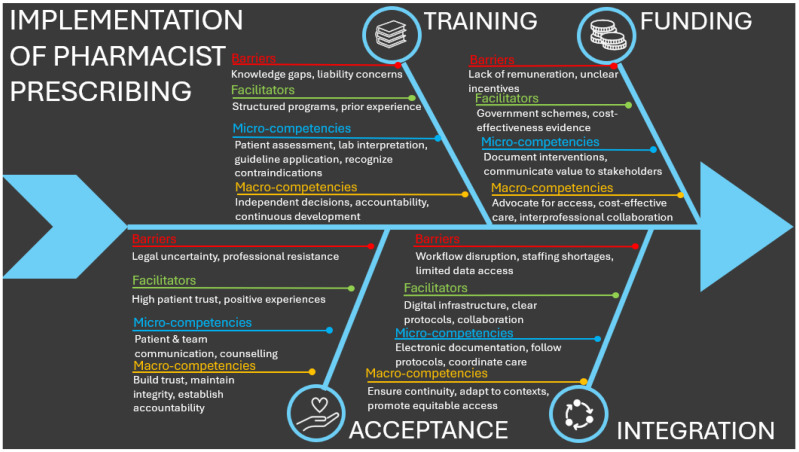
Fishbone diagram synthesizing barriers, facilitators, and inferred competencies for pharmacist prescribing, structured across four key dimensions.

The synthesis of barriers, facilitators, and inferred competencies is presented in a fishbone diagram (Figure 5), which outlines four key dimensions shaping pharmacist prescribing: training, funding, acceptance, and integration. Each dimension contains barriers that are counterbalanced by facilitators, while micro- and macro-competencies are positioned as critical for safe and effective prescribing. Together, these interlinked factors illustrate the readiness and feasibility of pharmacist prescribing, offering a structured framework for implementation.

## 4. Discussion

This systematic review examined international roles and inferred competencies in community pharmacist prescribing. It also identified contextual barriers and facilitators, with the secondary aim of informing implications for other healthcare systems considering implementation.

International roles of community pharmacists in prescribing were increasingly recognized as an essential component of primary care delivery. In total, 23 studies (2015–2024) were included and demonstrated a progressive global trend toward expanding the scope of pharmacist-led prescribing, albeit with marked variability in training, funding, acceptance, and integration frameworks. The findings affirm that pharmacist prescribing—particularly for minor ailments, contraception, chronic disease management, and acute conditions—has been successfully operationalized in countries such as Canada and selected US states, with documented evidence of clinical safety, efficiency, and high patient satisfaction [21,23,34,35,36]. Across these settings, pharmacists perform a wide range of tasks, including patient assessment, point-of-care testing, interpretation of laboratory results, and initiation or modification of treatment plans, reflecting an expanded clinical role that integrates independent decisions, accountability, and interprofessional collaboration [10,23,36,38,40].

Legal and regulatory frameworks emerge as critical determinants of pharmacists’ prescribing roles. National regulations define the scope of authority, eligibility criteria, and permitted medications, distinguishing independent prescribing from protocol- or collaboration-based models. Temporary pandemic exemptions further demonstrated regulatory flexibility under emergency conditions [32,33].

The implementation of pharmacist prescribing is further shaped by a range of facilitators and barriers at organizational, professional, and societal levels. Identified facilitators included government support, clinical training, interprofessional collaboration, and access to patient records, which enhance confidence, accountability, and the safe delivery of care [27,34,36]. Conversely, barriers include knowledge gaps, limited diagnostic tools, restricted access to patient records, inadequate remuneration, workflow disruptions, and unclear role perception among patients and physicians, which can impede optimal implementation and affect patient care outcomes [4,20,23,29,32,35]. Comparable challenges—particularly insufficient training in diagnostic competencies, weak regulatory backing, and inadequate funding—have also been reported in earlier reviews [6,44].

Building on the identified roles, this review then derived the competencies required for pharmacist prescribing. These competencies were intentionally simplified to serve as a practical orientation for community practice rather than a formal assessment framework. They highlight essential micro-competencies (patient assessment, documentation, communication, therapy monitoring) and macro-competencies (professional judgment, accountability, adaptability, and advocacy) as situated within the broader social and professional context of prescribing. Despite their simplicity, these competencies align closely with established international frameworks. For example, the Lebanese Specialized Competencies Framework for Community Pharmacists (SCF-CP) [45] maps micro-competencies to domains of fundamental skills, safe medicine use, and professional skills, while macro-competencies correspond to domains such as public health, pharmacy management, and emergency preparedness. Similarly, Australia’s “Prescribing Competencies Framework: Embedding Quality Use of Medicines into Practice” (2021) distinguishes micro-level prescribing skills from macro-level professional behaviours, including reflective practice and interprofessional collaboration [46].

Regulatory restrictions currently limit independent prescribing in many countries; however, exceptions such as emergency medication supply (Austria, Poland) and supervised hospital therapy adjustments (Austria) offer opportunities for pilot implementation [11,12,47]. Addressing barriers, including limited patient record access, insufficient interprofessional collaboration, and a lack of diagnostic tools, will be essential for meaningful adoption. Developed inferred competencies, distinguishing micro- and macro-competencies, can guide the development of national standards for pharmacist prescribing and serve as an international reference point for countries seeking to introduce similar roles. Surveys indicate high willingness among pharmacists to take on expanded responsibilities, supported by training and infrastructure, suggesting readiness for greater clinical roles [12].

### Strengths and Limitations

This review adhered to rigorous methodological standards, including PROSPERO registration, PRISMA 2020 compliance, and dual independent screening and appraisal using CASP tools, with inter-rater reliability assessed via Cohen’s Kappa. Limitations include restriction to English-language studies, exclusion of grey literature and regulatory documents, and a search limited to three databases (PubMed, CINAHL, Cochrane), which may have omitted relevant evidence. Heterogeneity in study designs and outcomes precluded meta-analysis, necessitating a narrative synthesis.

Most included studies were cross-sectional or descriptive with small, non-randomized samples, limiting generalizability. Reliance on pharmacist self-reporting may introduce response bias [22,29], and patient perspectives were largely absent. Only two studies directly compared pharmacist- versus physician-led care [35,36], highlighting the need for more robust outcome-based research. Inconsistent terminology for “prescribing” and the predominance of studies from high-income countries, particularly Canada and the US, further limit cross-national applicability. It was noted that several drugs newly authorized for pharmacist prescribing in the United States or Canada were already available as over-the-counter medications in European pharmacies (omeprazole, triptanes, emergency contraception). Moreover, differences in regulatory frameworks, training programs, and clinical contexts underscore that results may not be universally generalizable. Moreover, differences in regulatory frameworks, training programs, and clinical contexts underscore that results may not be universally generalizable.

The search strategy was restricted to terms related to “pharmacist” and “prescribing”, which may have led to the omission of studies addressing pharmacist involvement in broader care models or multi-component interventions in which prescribing represented one element (i.e., pharmacist care). As a result, studies focusing on condition management or collaborative care approaches that included pharmacist prescribing might not have been captured. Furthermore, a citation search, including forward and backward bibliography screening, was not undertaken. The absence of this step may have limited the comprehensiveness of the search and reduced the likelihood of identifying relevant studies published in journals not indexed within the selected databases.

Despite these limitations, the review offers a structured thematic synthesis of 23 international studies, integrating comparative models and real-world implementation insights, thereby supporting the translation of findings into policy and practice.

## 5. Conclusions

This systematic review provides evidence that community pharmacist prescribing has evolved beyond an experimental approach to become a validated, safe, and effective element of primary care, provided it operates within well-defined regulatory frameworks and is supported by comprehensive training and systemic integration. Empirical evidence from countries with mature systems demonstrates that pharmacists are capable of delivering high-quality prescribing services for minor ailments, contraception, chronic disease management, and acute conditions, consistently yielding favorable clinical outcomes and high levels of patient satisfaction. Nonetheless, progress in this domain remains inconsistent. Regions lacking robust legal frameworks, sustainable reimbursement mechanisms, and access to patient health records risk underutilizing pharmacists’ expertise, thereby perpetuating gaps in care, especially in rural and underserved areas. The competencies identified in this review offer a practical framework for establishing national standards and can inform phased policy development. By harmonizing regulatory authority, competency development, and interprofessional collaboration, pharmacist prescribing has the potential to significantly alleviate physician workload, enhance continuity of care, and improve equitable access to medications. The existing evidence base supports broader implementation, positioning pharmacist prescribing as a key strategy to strengthen primary care capacity amid increasing workforce shortages and rising healthcare demands.

## Figures and Tables

**Table 1:** Search strategy used for each database.

Database	Search Date	Search String (Descriptors & Boolean Operators)
PubMed	20 January 2025	((community AND pharmacist AND prescribing AND service AND pharmacy) NOT hospital)
CINAHL (EBSCOhost)	21 January 2025	((community AND pharmacist AND prescribing AND service AND pharmacy) NOT hospital)
Cochrane Library (Ovid)	22 January 2025	((community AND pharmacist AND prescribing AND service AND pharmacy) NOT hospital)

**Table 2:** Data summary of the included studies.

Authors, Year	Country	Study Design	Focus and Key Area	Population/Medication(s)	Barriers (−)/Facilitators (+)
Mansell et al., 2015 [34]	Canada (Saskatchewan)	Cross-sectional survey	Patient-reported outcomes (incl. satisfaction, and health-seeking) after pharmacist prescribing for minor ailments Key areas: Current role of community pharmacists; prescribing settings and models; barriers and facilitators	Community pharmacists Medication(s): Various prescription agents for minor ailments (e.g., antiviral creams, antihistamines, antibiotics)	− Lack of feedback for most events, limited capture of minor ailment prescriptions + High patient satisfaction with symptomatic improvement and service quality, with most patients trusting pharmacists
Tsuyuki et al., 2016 [36]	Canada (Alberta)	Multicenter RCT	Cardiovascular risk management (pharmacist-led case finding, prescribing, and test-and-treat interventions) Key areas: Current role; Prescribing settings and models; Barriers and facilitators)	Community pharmacists (n = 56 sites), patients at high cardiovascular risk (n = 723) Medication(s): Antihypertensives, lipid-lowering agents, antidiabetics, smoking cessation aids	− Short follow-up as targeted outcomes [Low-Density Lipoprotein Cholesterol (LDL-C), blood pressure, HbA1c, sustained smoking cessation] require longer to show full effects. + Improved clinical outcomes (significantly reduced cardiovascular risk and improved control of blood pressure, lipids, glycemic status, and smoking cessation); pharmacist training program
Bachyrycz et al., 2017 [25]	United States (New Mexico)	Registry analysis	Implementation of naloxone prescribing Key areas: Regulatory framework; prescribing settings and models; barriers and facilitators	Community pharmacists certified under the Naloxone Pharmacist Prescriptive Authority Program (n = 196); patients at risk of opioid overdose [n = 133 reported Naloxone Rescue Kit (NRK) prescriptions] Medication(s): Naloxone (intranasal, via NRK)	− Rural access, limited pharmacist certification, stigma, reimbursement + Direct access, patient engagement
Ung et al., 2017 [38]	Australia (Western Australia)	Quantitative cross-sectional survey using case vignette methodology	Prescribing appropriateness for infections (prescribing oral antibiotics) Key areas: Current role; Prescribing settings and models; Barriers and facilitators	Community pharmacists (n = 82); 425 case vignette responses across various infection types Medication(s): Amoxicillin, trimethoprim, flucloxacillin, cephalexin, amoxicillin/clavulanic acid	− Diagnostic uncertainty (specifically in complex infections), training needs + High prescribing confidence for common infections: Urinary Tract Infection (UTIs), cellulitis, and acne
Gauld et al., 2017 [39]	New Zealand	Before-and-after study	Trimethoprim supply in women with uncomplicated cystitis and stewardship Key areas: Regulatory framework; prescribing settings and models; barriers and facilitators	Community pharmacists (n = 139 pharmacies pre, n = 120 post), women aged 16–65 with uncomplicated cystitis Medication(s): Trimethoprim, nitrofurantoin, norfloxacin, ciprofloxacin	− Low uptake, public awareness gaps + Guideline-conformant prescribing; effective stewardship
Batra et al., 2018 [26]	United States (California)	Telephone mystery shopper survey	Access to hormonal contraception Key areas: regulatory framework; prescribing settings and models; barriers and facilitators	Retail pharmacies in California (n = 457), stratified by rurality and pharmacy type Medication(s): Hormonal contraceptives (pill, patch, ring, injection)	− Low pharmacists’ service participation, unclear incentives, variable implementation fidelity + Pharmacists‘ protocol compliance
Dingman et al., 2018 [27]	United States (50 states + District of Columbia)	Legal analysis using the Centers for Disease Control and Prevention’s (CDC) Public Health Law Program database and standardized coding algorithm	HPV vaccine authority across states Key areas: Regulatory framework; Barriers and facilitators	Not applicable (legal jurisdictions) Medication(s): HPV vaccine	− Age restrictions (only 22 states permitted vaccination of 11–12-year-olds, many laws imposed age restrictions or required prescriber involvement, limiting access) + Legal access in many states: n = 5 allowed prescriptive authority (no third party), n = 32 allowed general third-party authorization, and n = 3 required patient-specific authorization
Schwartzberg et al., 2018 [42]	Israel (with international comparison)	Policy review	Comparison of pharmacist service models (Israel vs. International) Key areas: Current role of community pharmacists; prescribing settings and models; barriers and facilitators	Not applicable Medication(s): Emergency supply, statins, antihypertensives, inhaled corticosteroids, and vaccines	− Lack of time, insufficient remuneration, limited access to medical records + Expanded legal authority in Israel (prescribing, vaccination, and emergency dispensing)—aligning with international trends
Anderson et al., 2019 [24]	United States (Oregon)	Retrospective Medicaid claims analysis	Evaluation of contraceptive pharmacist-led prescribing Key areas: Prescribing settings and models, Regulatory framework	community pharmacists (n = 162) Medication(s): combined oral contraceptive pills, progestin-only pills, transdermal patches	− Racial access disparities + High reach among new users
Vu et al., 2019 [29]	United States (California)	Cross-sectional survey	Readiness to prescribe contraception Key areas: Current role; Barriers and facilitators	Community pharmacists in California (n = 121) Medication(s): hormonal contraceptives	− Time constraints, liability concerns, lack of reimbursement + High comfort and intent with clinical tasks such as identifying contraindications and providing patient education.
Rafie et al., 2019 [20]	United States (California)	Semi-structured interviews	Pharmacists’ perspectives on prescribing hormonal contraception prior to statewide protocol implementation. Key areas: Current role of community pharmacists; regulatory framework; barriers and facilitators	Community pharmacists (n = 30) from urban, suburban, and rural settings Medication(s): hormonal contraceptives (pill, patch, ring, injection)	− Knowledge gaps, religious objections, limited private space, reimbursement issues, liability concerns. + Public health benefit of expanded access and willingness to participate under appropriate conditions
Gomez et al., 2020 [28]	United States (California)	Structured telephone interviews	Implementation in independent pharmacies Key area: Barriers and facilitators	Pharmacists (n = 36) from independent pharmacies Medication(s): Hormonal contraceptives	− Lack of reimbursement, business risks, liability concerns, and time/resource limitations impeded implementation. + Support of the expansion of roles and high potential to increase access.
Stone et al., 2020 [22]	United States (21 states)	Cross-sectional survey	Training needs for contraception prescribing Key areas: Current role of community pharmacists; barriers and facilitators	Pharmacists in 21 US states (n = 823) Medication(s): Hormonal contraceptives (pill, patch, ring, injection)	− Inadequate curriculum coverage, limited familiarity with clinical guidelines, and preference for additional training formats + Higher confidence with experience or residency training and readiness to prescribe
Herman et al., 2020 [30]	United States (New Mexico)	Semi-structured telephone interviews	Rural pharmacists’ readiness in prescribing contraception Key areas: Current role of community pharmacists; regulatory framework	Rural community pharmacists (n = 21) from diverse regions across New Mexico Medication(s): Hormonal contraceptives (pill, patch, ring, injection)	− Insufficient training, lack of reimbursement, and liability concerns, with limited support infrastructure in rural practice settings. + Pharmacists recognized their accessibility and trusted rural role, expressing willingness to expand contraception prescribing.
Spann et al., 2020 [23]	United States (Idaho)	Pilot study	Implementation and patient acceptance of pharmacist-led statin prescribing for type 2 diabetes. Key areas: Current role of community pharmacists; regulatory framework; prescribing settings and models; barriers and facilitators	Community pharmacists in four Albertsons pharmacies, patients with type 2 diabetes eligible for statin therapy Medication(s): moderate-intensity statins	− Difficulty contacting patients, delays due to mail-in lab tests, and a lack of integration with electronic health records. + Positive patient perception and successful implementation
Woodill and Bodnar, 2020 [37]	Canada (Nova Scotia)	Evaluation of Community Pharmacist-led Anticoagulation Management Service based on qualitative and quantitative data	Model evaluation based on Point-of-care International Normalized Ratio (INR) test, assessment and dosage adjustment prescribing, counselling and providing support in adherence tools Key areas: Current role of community pharmacists; barriers and facilitators	Community Pharmacists (n = 106), patients (n = 946), primary care providers (n = 237; physicians = 225, nurse practitioners = 12) Medication(s): Warfarin, Novel Oral Anticoagulants (NOACs)	− Manufacturer test strip error (was not identified) + Effective prescribing model; improved time in therapeutic range outcomes for patients. In addition, cost-effective solution for health systems
Zimmermann et al., 2021 [41]	Poland	Retrospective data analysis	COVID-related expansion of prescribing from emergency-only to broad pharmacist prescribing Key areas: Current role of community pharmacists; regulatory framework; prescribing settings and models; barriers and facilitators	Pharmacists from community pharmacies (n = 842) and national prescribing dataset (n = 18,529 prescriptions Medication(s): Cardiovascular, respiratory, dermatological, alimentary tract, nervous system, anti-infectives	− Lack of reimbursement, unclear legal definitions of “health risk” limited practical implementation + Expanded legal access across various conditions including chronic diseases and minor ailments, significantly increasing access
Bishop & Rosenberg-Yunger, 2022 [33]	Canada	Semi-structured interviews	Examination of Canadian pharmacists’ use of an emergency exemption to provide opioid agonist therapy during COVID-19 Key areas: Current role of community pharmacists; regulatory framework; prescribing settings and models; barriers and facilitators	Community and primary care pharmacists (n = 19) who used the Controlled Drugs and Substances Act exemption Medication(s): Opioid agonist therapy (e.g., methadone, buprenorphine/naloxone, hydromorphone)	− Stigma, lack of training and infrastructure, time burden, and variability in pharmacy willingness to provide opioid use disorder services. + Improved continuity of care, facilitate harm reduction, and expand access through clinical assessments, prescription transfers, and emergency supplies.
Grant et al., 2023 [32]	Canada (Nova Scotia)	Cross-sectional survey	Prescribing changes during COVID-19 Key areas: prescribing settings and models; barriers and facilitators	Community pharmacists (n = 190) prescribing, Medication(s): Antibiotics, hormonal contraceptives, antifungals, antivirals, antihistamines, smoking cessation aids, Gastroesophageal Reflux Disease (GERD) treatments, acne therapy, vaccines	− Lack of remuneration, staffing shortages, and workflow constraints + Increase in prescribing volume particularly for government-funded services and in categories like renewals and uncomplicated cystitis.
Grant et al., 2023 [31]	Canada (Nova Scotia)	Cohort study based on health data	Prescribing trends and access over 3 years Key areas: current role of community pharmacists; prescribing settings and models; barriers and facilitators	Community pharmacists in Nova Scotia (n = 1182); patient cohort (n = 372.203) Medication(s): GERD treatments, vaccines, contraceptives, antibiotics, smoking cessation aids	− Socioeconomic gaps, lower uptake in urban/high-income areas, logistical challenges related to reimbursement and lab integration. + Increased prescribing; high uptake for approved conditions such as GERD, vaccination, and contraception; care gaps in underserved populations.
Laopaiboonkun et al., 2024 [40]	Thailand	Cross-sectional survey	Guideline adherence in UTI prescribing Key areas: Current role of community pharmacists; prescribing settings and models; barriers and facilitators	Community pharmacists (n = 349) Medication(s): ciprofloxacin, norfloxacin, ofloxacin, amoxicillin/clavulanate, nitrofurantoin, fosfomycin	− Diagnostic confusion in distinguishing between complicated and uncomplicated cystitis, highlighting diagnostic gaps, especially, especially in older pharmacists + Strong guideline adherence
Azad et al., 2024 [21]	United States (California—Central Valley)	Mixed-methods study	Contraception access in rural areas Key areas: Prescribing settings and models; barriers and facilitators	Community pharmacists in 11 Central Valley counties (n = 576 pharmacies contacted, n = 75 furnishing) Medication(s): hormonal contraceptives (oral contraceptives)	− Lack of reimbursement, low public awareness, limited pharmacist certification, and time/staffing constraints + High accessibility, privacy, cost-effectiveness
Sandhu et al., 2024 [35]	Canada (Alberta)	RCT	Evaluation of anticoagulant prescribing in atrial fibrillation Key areas: Current role of community pharmacists; prescribing settings and models; barriers and facilitators	Community pharmacies (n = 27); patients with untreated or undertreated atrial fibrillation (n = 80) Medication(s): Oral anticoagulants (e.g., warfarin, apixaban, rivaroxaban)	− Recruitment and physician resistance, logistical constraints such as the COVID-19 pandemic + Increased guideline-concordant anticoagulant, better patient adherence, satisfaction, guideline use

**Table 3:** Inferred Competencies.

Role	Legal/Regulatory Framework	Micro-Competency (Professional Skills)	Macro-Competency (Person-Level, Overarching)	Role Context/Performance (Professional Excellence in Social Context)
**Clinical Role Expansion**	Defined by national regulations (independent vs. protocol-based prescribing) Mandatory training/qualification Emergency/pandemic exemptions may temporarily expand authority [32,33,39,41,42]	Conduct patient assessments Conduct point-of-care tests Interpret lab results Apply evidence-based guidelines Identify contraindications [23,36,38,40]	Assess, diagnose, and manage patient therapy Initiate or modify treatment plans Monitor outcomes [23,30,35,36,37]	Role involves exercising independent clinical judgment, collaborating effectively with healthcare teams, and adapting practices to dynamic patient and organizational contexts, demonstrating professional excellence and accountability
**Public Health and Accessibility**	Regulations determine pharmacists’ role in underserved/rural areas Temporary COVID-19 measures enabled broader prescribing authority [21,25,30,31,34,41,42]	Issue prescriptions (Provide vaccinations) Implement harm-reduction strategies Educate patients on adherence Counsel on minor ailments [3,21,27,30,34,36]	Ensure patient access to care Maintain continuity of therapy and care Deliver preventive services Manage minor/chronic conditions Reduce harm [23,25,31,33,34,41]	Role requires balancing public health priorities with patient safety, promoting equitable access, and contributing to community health, enacting professional responsibility in societal contexts
**Readiness and Self-Perception**	Training, regulatory support, and a clearly defined scope influence confidence, accountability, and legal liability in prescribing. [20,22,29,31]	Document interventions (electronically) Recognize limitations Follow guidelines and/or protocols Communicate effectively with patients and healthcare teams Pursuing continuous professional development [20,30,37,39,40,41]	Make independent clinical decisions safely Engage in reflective practice Maintain professional accountability [20,23,31]	Role emphasizes safe and accountable prescribing, reflective practice, and continuous adaptation to evolving professional responsibilities, reinforcing trust and professional integrity within social and healthcare contexts

## Data Availability

No new data were created or analyzed in this study. Data sharing is not applicable to this article.

## Supplementary Materials

The following supporting information can be downloaded at: https://www.mdpi.com/article/10.3390/pharmacy13060157/s1, File S1: PRISMA 2020 Checklist.

## Abbreviations

The following abbreviations are used in this manuscript:

CASP	Critical Appraisal Skills Programme
CDC	Centers for Disease Control and Prevention
GERD	Gastroesophageal Reflux Disease
GPs	General Practitioners
HbA1c	Glycosylated Hemoglobin
HPV	Human Papillomavirus
INR	International Normalized Ratio
LDL-C	Low-Density Lipoprotein Cholesterol
NOAC	Novel Oral Anticoagulant
NRK	Naloxone Rescue Kit
PRISMA	Preferred Reporting Items for Systematic Reviews and Meta-Analyses
PROSPERO	International Prospective Register of Systematic Reviews
RCT	Randomized Controlled Trial
UTI	Urinary Tract Infection

## Appendix A. Risk of Bias Assessment Raw Data

**Table A1:** Summary of the CASP-Based Quality Appraisal of the 23 Included Studies.

Author, Year	CASP-Tool	Raw Score	Score (%)	Quality Rating
[22]	Descriptive/Cross-Sectional Studies	11/11	100	High quality
[38]	Descriptive/Cross-Sectional Studies	11/11	100	High quality
[41]	Descriptive/Cross-Sectional Studies	11/11	100	High quality
[31]	Descriptive/Cross-Sectional Studies	10.5/11	95.5	High quality
[29]	Descriptive/Cross-Sectional Studies	10.5/11	95.45	High quality
[26]	Descriptive/Cross-Sectional Studies	10.5/11	95.45	High quality
[33]	Qualitative Research	9.5/10	95	High quality
[37]	Cohort Studies	13/14	92.86	High quality
[32]	Descriptive/Cross-Sectional Studies	10/11	90.91	High quality
[28]	Qualitative Research	9/10	90	High quality
[20]	Qualitative Research	9/10	90	High quality
[40]	Descriptive/Cross-Sectional Studies	9.5/11	86.36	High quality
[35]	Randomized Controlled Trials (RCTs)	11/13	84.6	High quality
[30]	Qualitative Research	8/10	80	High quality
[39]	Descriptive/Cross-Sectional Studies	8.5/11	77.27	Moderate quality
[23]	Descriptive/Cross-Sectional Studies	8.5/11	77.27	Moderate quality
[36]	Randomized Controlled Trials (RCTs)	10/13	76.92	Moderate quality
[24]	Cohort Studies	10.5/14	75	Moderate quality
[25]	Descriptive/Cross-Sectional Studies	8/11	72.73	Moderate quality
[34]	Descriptive/Cross-Sectional Studies	7/11	63.64	Moderate quality
[27]	Systematic reviews with meta-analysis of observational studies	6/10	60	Low quality
[21]	Descriptive/Cross-Sectional Studies	6/11	54.5	Low quality
[42]	Qualitative Research	3.5/10	35	Low quality

**Table A2:** Interrater Reliability for a Subsample of 19 out of 23 Included Studies, Assessed Using Cohen’s Kappa and Interpreted According to Landis and Koch (1977) [18].

Author, Year	Checklist Items	Cohen’s Kappa	Interpretation
[22]	10	1	Almost perfect
[24]	10	1	Almost perfect
[33]		1	Almost perfect
[42]	10	1	Almost perfect
[41]	11	1	Almost perfect
[31]	11	1	Almost perfect
[39]	11	1	Almost perfect
[26]	11	1	Almost perfect
[23]	11	1	Almost perfect
[40]	11	1	Almost perfect
[21]	11	1	Almost perfect
[38]	11	0.48	Moderate
[20]	10	0.47	Moderate
[25]	11	0.41	Moderate
[36]	13	0.31	Fair agreement
[29]	11	0.29	Fair agreement
[32]	11	0,29	Fair agreement
[35]	13	0.29	Fair agreement
[34]	11	0.22	Fair agreement
